# A three-dimensional analysis of the morphological evolution and locomotor behaviour of the carnivoran hind limb

**DOI:** 10.1186/1471-2148-14-129

**Published:** 2014-06-14

**Authors:** Alberto Martín-Serra, Borja Figueirido, Paul Palmqvist

**Affiliations:** 1 Departamento de Ecología y Geología, Facultad de Ciencias, Universidad de Málaga, Campus de Teatinos s/n, 20971 Málaga, Spain

**Keywords:** Carnivora, Hind limb, Allometry, Locomotion, Phenotypic evolution, Convergence

## Abstract

**Background:**

The shape of the appendicular bones in mammals usually reflects adaptations towards different locomotor abilities. However, other aspects such as body size and phylogeny also play an important role in shaping bone design.

We used 3D landmark-based geometric morphometrics to analyse the shape of the hind limb bones (i.e., femur, tibia, and pelvic girdle bones) of living and extinct terrestrial carnivorans (Mammalia, Carnivora) to quantitatively investigate the influence of body size, phylogeny, and locomotor behaviour in shaping the morphology of these bones. We also investigated the main patterns of morphological variation within a phylogenetic context.

**Results:**

Size and phylogeny strongly influence the shape of the hind limb bones. In contrast, adaptations towards different modes of locomotion seem to have little influence. Principal Components Analysis and the study of phylomorphospaces suggest that the main source of variation in bone shape is a gradient of slenderness-robustness.

**Conclusion:**

The shape of the hind limb bones is strongly influenced by body size and phylogeny, but not to a similar degree by locomotor behaviour. The slender-robust “morphological bipolarity” found in bone shape variability is probably related to a trade-off between maintaining energetic efficiency and withstanding resistance to stresses. The balance involved in this trade-off impedes the evolution of high phenotypic variability. In fact, both morphological extremes (slender/robust) are adaptive in different selective contexts and lead to a convergence in shape among taxa with extremely different ecologies but with similar biomechanical demands. Strikingly, this “one-to-many mapping” pattern of evolution between morphology and ecology in hind limb bones is in complete contrast to the “many-to-one mapping” pattern found in the evolution of carnivoran skull shape. The results suggest that there are more constraints in the evolution of the shape of the appendicular skeleton than in that of skull shape because of the strong biomechanical constraints imposed by terrestrial locomotion.

## Background

One of the key aspects of species biology is locomotion, which determines many important behavioural activities such as foraging, hunting, escaping from predators, or migrating [1-3]. Therefore, the study of locomotor adaptations in living and extinct species is crucial to understanding their role in present and past ecosystems [4,5].

Natural selection has led to morphological adaptations in the postcranial skeleton, which have been largely treated in the literature as “ecomorphological indicators” of locomotion modes in living species. Thus, several studies on locomotor evolution in mammals have used limb indicators of ecological adaptations to determine paleobiological aspects in extinct species [6-14]. However, natural selection is not always the only factor in shaping morphological traits [15-17]. It is important to investigate the effects of different potential sources of variation prior to identifying possible ecomorphological correlates, such as phylogenetic inheritance [15-17] or allometry [18-23].

This study investigated the influence of phylogeny, allometry, and locomotor behaviour in shaping the morphology of the hind limb bones (i.e., femur, tibia, and pelvic girdle bones) in mammalian carnivores (extant and extinct taxa from the order Carnivora plus some taxa from the closely-related order Creodonta). We used mammalian fissiped carnivorans (i.e., a paraphyletic group that includes members of the mammalian order Carnivora exclusive of members of the clade Pinnipedia, which were excluded due to their highly aquatic specialization) as a model system for the following reasons: *(i)* their mode of locomotion is remarkably diverse, including arboreal, terrestrial, and semiaquatic modes [24-28]; *(ii)* they have a different hunting styles, including pursuing, pouncing, ambushing, or hunting [9,29-36]; and *(iii)* their phylogenetic relationships are well characterised [37].

This article forms part of a wider study on the ecomorphology and evolution of the appendicular skeleton in the order Carnivora with a particular focus on the influence of various factors in shaping the fore- and hind limb bones. We complement the analysis of the forelimb [38] by studying the evolution of the hind limb. This study will therefore lead to a complete picture of the morphological evolution of all major limb bones of the carnivoran appendicular skeleton as a whole.

Our predictive hypothesis was that there would be many similarities between the evolution of the bone shape of the fore- and hind limbs. However, as these limbs have several functional differences and anatomical peculiarities, we also predicted that there would be some differences in their patterns of evolution. For example, it has been demonstrated that the forelimbs of domestic dogs support a greater proportion of body weight than the hind limbs [39,40] and this could be the case for all fissiped carnivorans. If this supposition were correct, it would be reasonable to assume that allometry has less effect on the hind limb bones than on the forelimb bones. Furthermore, hind limbs are thought to be more important in providing impulse during acceleration and running than the forelimbs [39,41,42] and therefore locomotor behaviour could have a stronger influence on shaping the hind limb than the forelimb. On the other hand, many carnivoran species use their forelimbs for activities other than the ones involved in locomotion, such as grasping, climbing, or manipulating prey [28,33,43] and this could also be a potential source of morphological differences between the fore- and hind limbs.

We used 3D geometric morphometrics to characterize the morphology of the hind limb bones (i.e., femur, tibia and the pelvic girdle bones) in order to answer the following questions: *i*) Is there an allometric effect in shaping the morphology of the hind limb bones; *(ii)* Is there a phylogenetic signal in all hind limb bones? *(iii)* Is there an association between locomotor behaviour and the shape of these bones? *(iv)* What are the evolutionary pathways followed by the hind limb long bones? *(v)* Is the evolutionary pattern of the hind limb similar to that of the forelimb? *(vi)* Does the appendicular skeleton – fore- and hind limbs – reflect functional and ecological convergences similar to the way the craniodental skeleton reflects them?

## Results

### Phylogeny and size

**Figure 1:**
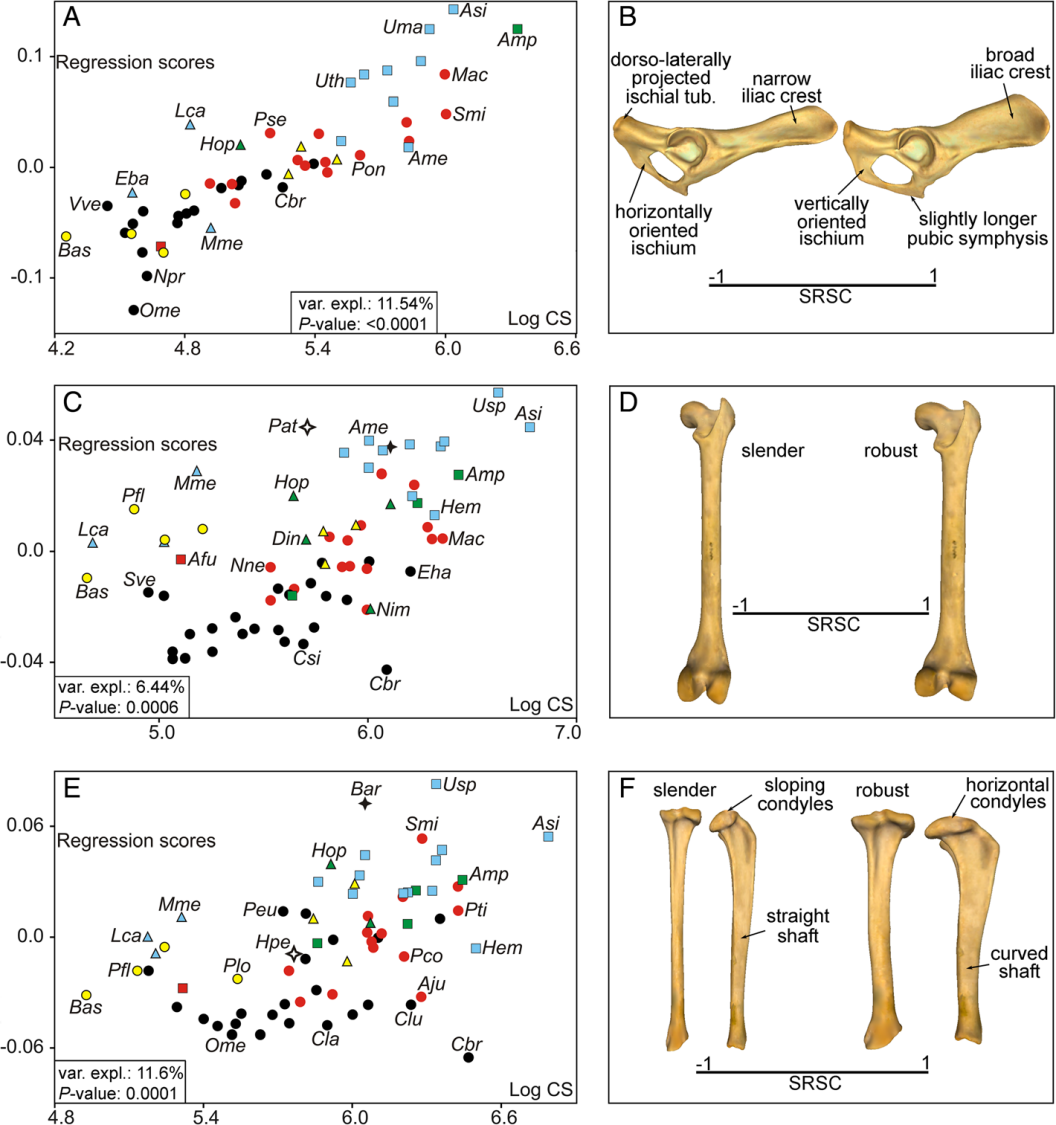
**Analysis of interspecific allometry.** Bivariate graphs derived from the multivariate regressions performed from the Pco against the Log-Cs for the pelvis **(A)**, femur **(C)**, and tibia **(E)**. The three-dimensional models showing the associated size-related shape change (SRSC) for the pelvis **(B [lateral view])**, femur **(D [caudal view])** and tibia **(F [caudal and lateral views])** are also shown. Symbols: red squares, Ailuridae; green squares, Amphicyonidae; black stars, Barbourofelidae; black circles, Canidae; empty stars, Creodonta; red circles, Felidae; yellow triangles, Hyaenidae; blue triangles, Mustelidae; green triangles, Nimravidae; yellow circles, Procyonidae; blue squares, Ursidae. See Additional file 3: Table S1 for the species labels. For the interactive three-dimensional shape models explained by size variation see Additional file 1.

**Figure 2:**
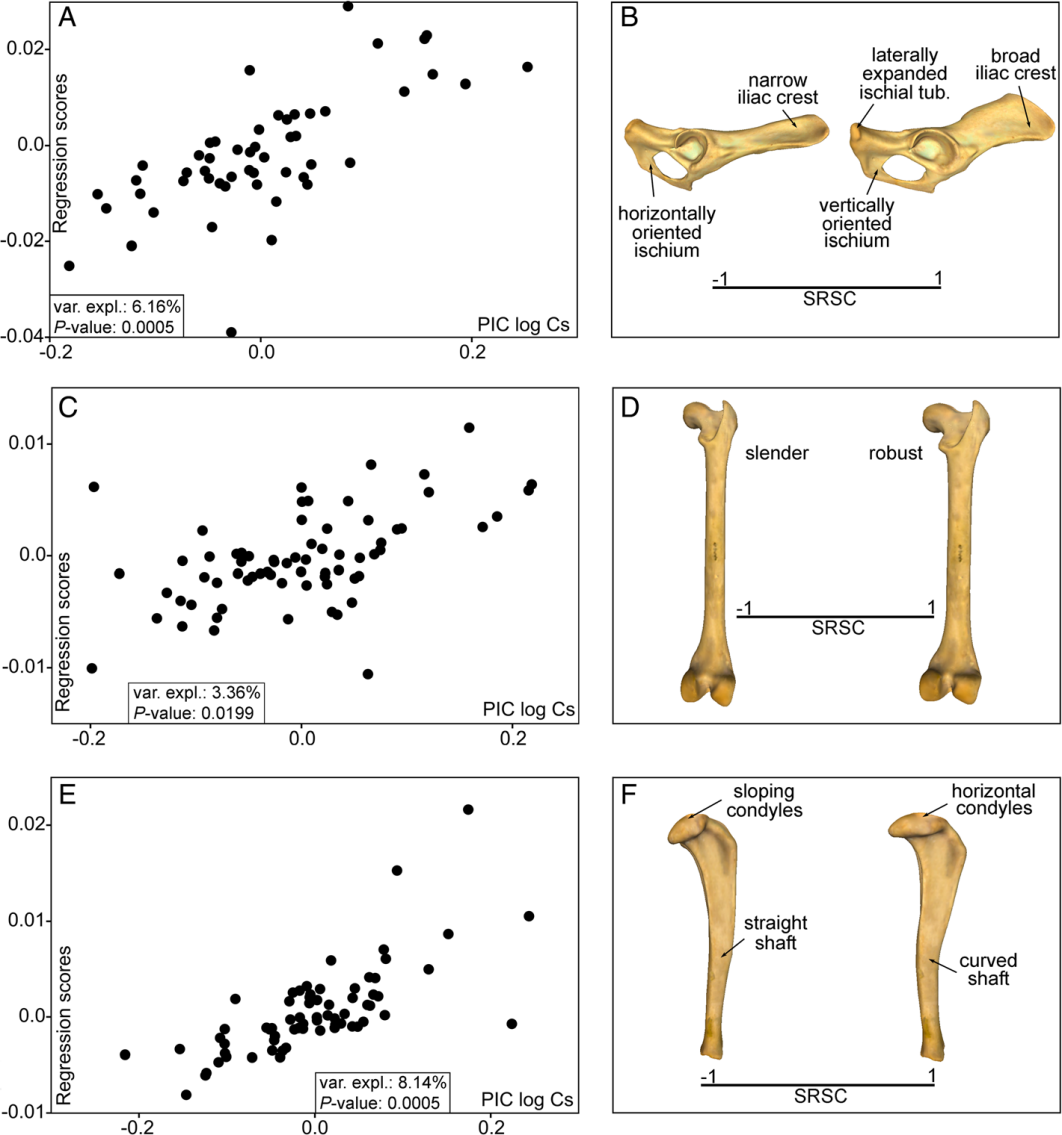
**Analysis of evolutionary allometry.** Bivariate graphs derived from the multivariate regressions performed from the contrasted Pco against the Log-CS, which has been adjusted through phylogenetic independent contrasts analysis, for pelvis **(A)**, femur **(C)**, and tibia **(E)**. The three-dimensional models showing the size-related shape change (SRSC) for the pelvis **(B [lateral view])**, femur **(D [caudal view])** and tibia **(F [lateral view])** are also shown.

The permutation tests performed to investigate the presence of a phylogenetic structure in shape (Procrustes coordinates, Pco) and size (Log-transformed centroid size, Log-Cs) showed statistically significant results for all the bones (Table 1). The multivariate regressions of shape on size were statistically significant in all cases and indicate the presence of interspecific allometry (Figure 1A, 1C and 1E). The shape changes explained by interspecific allometry are shown in Figure 1B, 1D, and 1F (also see Additional file 1).The multivariate regressions between the phylogenetic independent contrasts for shape on the contrasts for size also yielded significant results and suggest that allometry is not merely due to a phylogenetic effect (Figure 2A, 2C, and 2E). The shape changes explained by evolutionary allometry are shown in Figure 2B, 2D, and 2F.

**Table 1:** Results of assessing the presence of a phylogenetic signal in each hind limb bone shape (Pco) and size (Log-Cs)

**Bone**	**Shape**	**Size**
**Pelvis**	0.4635 (<0.0001)	4.2411 (<0.0001)
**Femur**	0.0764 (<0.0001)	4.0284 (<0.0001)
**Tibia**	0.0567 (<0.0001)	3.437 (<0.0001)

Numbers indicate the tree lengths obtained with each permutation test. The respective *p*-values are given between brackets.

### Phenotypic spaces and their histories of occupation

**Figure 3:**
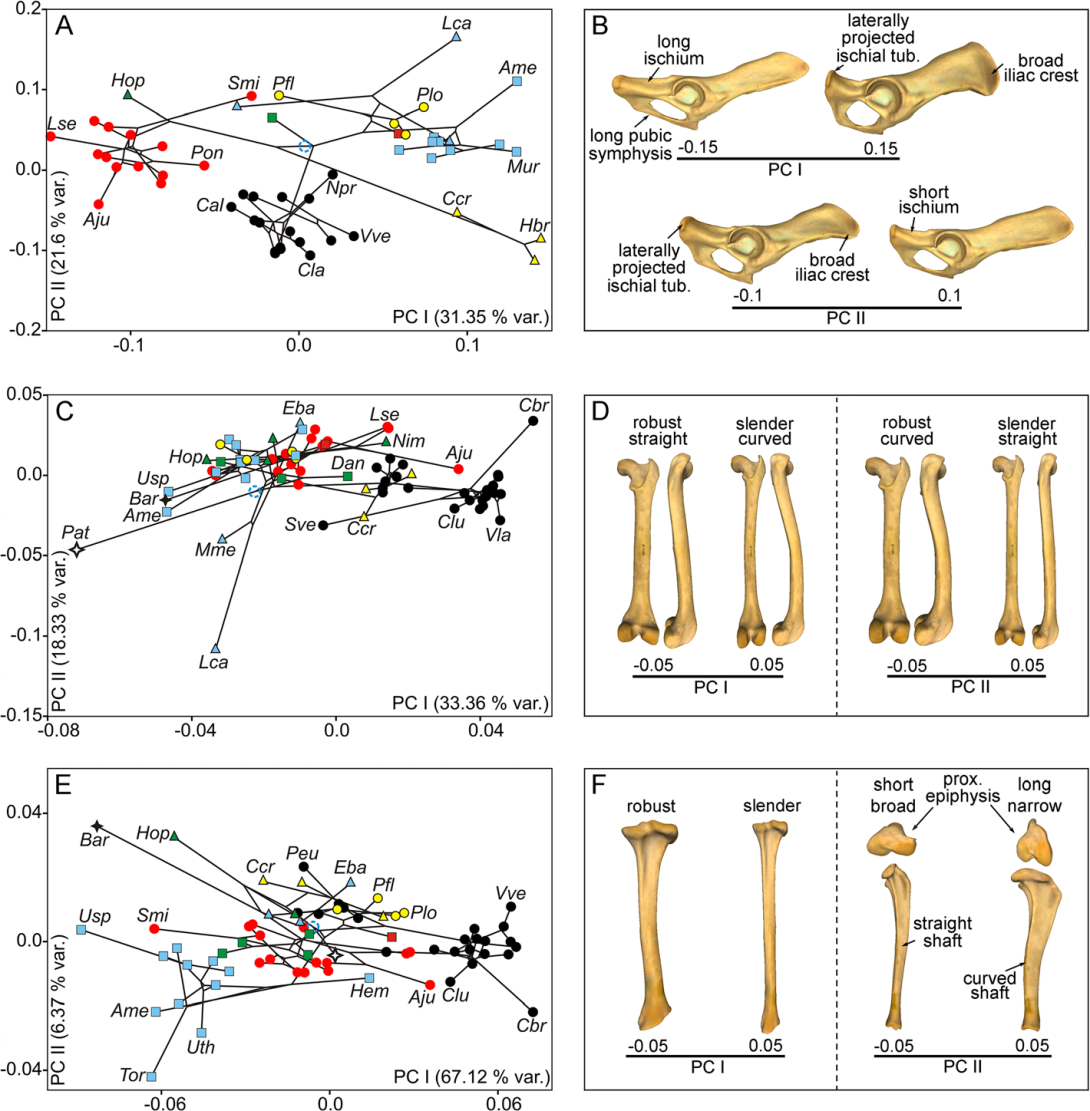
**Principal component analyses.** Bivariate graph derived from PC I and PC II with the regression residuals (Pco-Cs) for the pelvis **(A)**, femur **(C)**, and tibia **(E)**. The plots also show the tree topology mapped on the morphospace. Three-dimensional models showing the shape change associated with these axes for the pelvis **(B [lateral view])**, femur **(D [caudal and lateral views])**, and tibia **(F [caudal and proximal views])**. Blue empty circle: tree root; see Figure 3 for more symbols. See Additional file 2 for interactive models. See Additional file 3: Table S1 for species labels.

**Figure 4:**
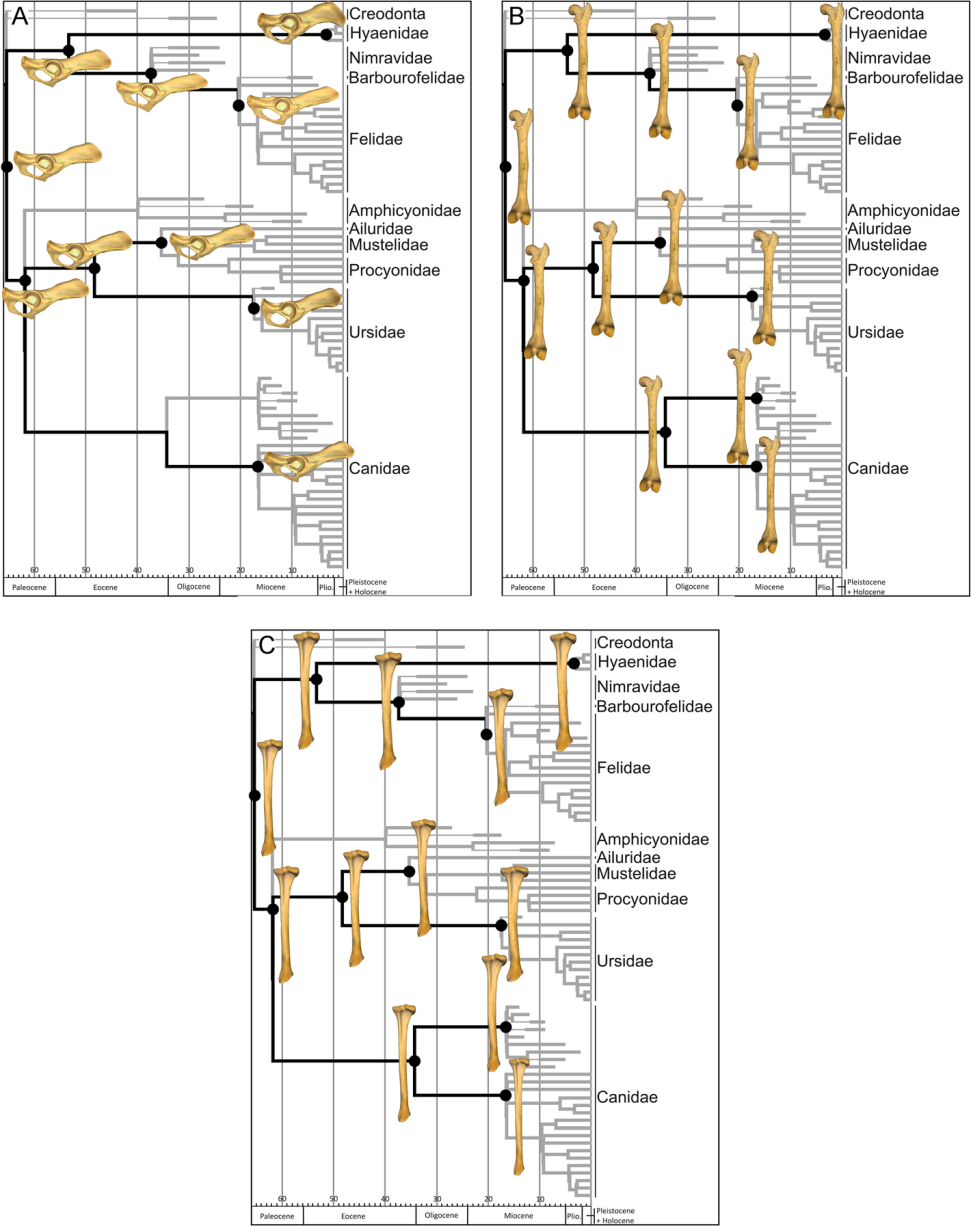
**Reconstruction of ancestral hind limb bone shape.** Pelvis **(A)**, femur **(B)** and tibia **(C)**. Three-dimensional models show the hypothetical morphology of the highlighted nodes (black circles).

Principal component analyses (PCA) were performed to investigate the morphological variability of each bone and their respective phylomorphospaces (Figure 3). The PCA performed on the shape of the pelvis (although we only analyzed one side of the pelvic girdle [innominate bone], we refer to it as the pelvic girdle or pelvis for easier understanding) provided two principal components (PC), which accounted for more than 52% of the original variance. The first PC (Figure 3A, *x* axis) differentiated the pelvis of hyaenids and ursids with positive scores from the shape of the pelvis of felids with negative scores. The second PC (Figure 3A, *y* axis) mainly differentiated musteloids (i.e., procyonids, ailurids, and mustelids) with positive scores from canids and hyaenids with negative scores. The corresponding shapes at the extremes of both eigenvectors are shown in Figure 3B and Additional file 2A.A visual inspection of this phylomorphospace shows that the terminal branches are relatively short and the internal branches are relatively long (Figure 3A). This pattern suggests that the pelvis shapes of closely related species are similar. This result was confirmed by the reconstruction of the pelvis shapes for the basal nodes of each family (Figure 4A) and shows that each family has a well-defined characteristic morphology.

The PCA performed on the shape of the femur yielded two significant PCs, which together explained around 52% of the original variance. The first PC (Figure 3C, *x* axis) mainly differentiated the extinct creodont *Patriofelis*, the ursids *Ailuropoda melanoleuca* and *Ursus spelaeus*, and the “false saber-tooth” *Barbourofelis* with negative scores from most canine canids. The maned wolf (*Chrysocyon brachyurus*) had extreme positive scores. In contrast, the second PC (Figure 3C, *y* axis) differentiated the species into a gradient that starts at the femur of *Eira barbara* (within mustelids), felids, and procyonids with positive scores and ends at the femur of *Lontra canadensis* with extreme negative scores. The corresponding shapes at the extremes of these eigenvectors are shown in Figure 3D and Additional file 2B.

The PCA performed on the shape of the tibia gave two significant PCs, which jointly accounted for approximately 74% of the total shape variation. PC I (Figure 3E, *x* axis) differentiated the tibia of most canines and *Acinonyx jubatus* (within felids) with positive scores from the tibia of *Barbourofelis*, *Hoplophoneus*, *Ursus spelaeus*, and *Ailuropoda melanoleuca* with extreme negative scores according to a set of morphological traits (Figure 3F and Additional file 2C). However, PC II (Figure 3E, *y* axis) differentiated the species into a gradient that starts at the tibia of *Barbourofelis* and *Hoplophoneus* and ends at the tibia of most ursids according to the shape changes shown in Figure 3F and Additional file 2C.The phylomorphospaces of the femur and tibia are clearly different from that of the pelvis (compare Figure 3A with Figure 3C and 3E) because the phylomorphospaces of the long bones have long terminal branches and short internal ones. This suggests that some families overlap with each other, creating a “messy” pattern. In fact, the reconstruction of the basal nodes of each family for both long bones suggests that shape divergence mainly occurred within each family (Figure 4B and 4C).

### Locomotor behaviour

A between-group PCA was performed for each bone to investigate the effect of locomotor behaviour on hind limb bone shapes (see Additional file 3: Table S1).

**Figure 5:**
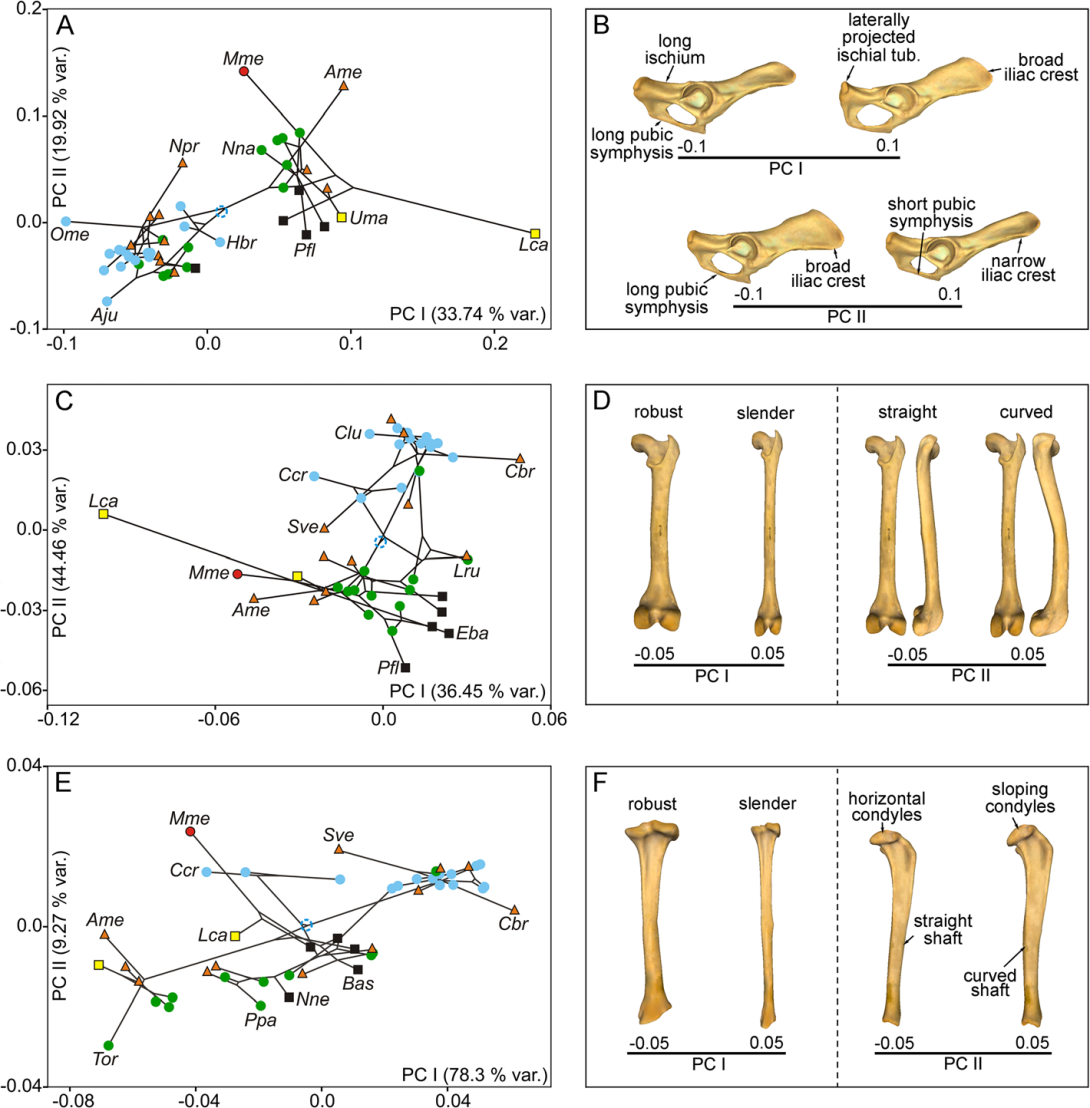
**Between-group principal component analyses.** Bivariate graph derived from PC I and PC II with the regression residuals (Pco-Cs) for pelvis **(A)**, femur **(C)**, and tibia **(E)**. The plots also show the tree topology mapped on the morphospace. Three-dimensional models showing the shape change associated with these axes for the pelvis **(B [lateral view])**, femur **(D [caudal and lateral views])**, and tibia **(F [caudal and lateral views])**. Symbols: blue empty circle, tree root; blue circles, cursorial; green circles, scansorial; orange triangle, terrestrial; black square, arboreal; yellow square, semiaquatic; red circle, semifossorial. See Additional file 4 for interactive models. See Additional file 3: Table S1 for species labels.

The first two PCs obtained for the pelvis explained around 54% of the total variance (Figure 5A). The first component mainly differentiated the Canadian river otter (*Lontra canadensis*) with positive scores from cursorial carnivores with negative scores (Figure 5A, *x* axis) according to a set of morphological traits (Figure 5B and Additional file 4A). However, the second component differentiated the semifossorial European badger (*Meles meles*) and the terrestrial giant panda (*Ailuropoda melanoleuca*) with positive scores from other species (Figure 5A, *y* axis; see Figure 5B and Additional file 4A for morphological changes). These PCs do not appear to clearly differentiate any of the other ecological groups.

The first two PCs obtained for the femur accounted for more than 80% of the total variance (Figure 5C). The first component differentiated semiaquatic *Lontra canadensis* with negative scores from other species with positive scores (Figure 5C, *x* axis). In contrast, the second component mainly differentiated cursorial species and some terrestrial species with positive scores from scansorial species, arboreal species, and some terrestrial species with negative scores (Figure 5C, *y* axis). The morphological changes associated with these eigenvectors are shown in Figure 5D and Additional file 4B.

The between-group PCA performed on the tibia provided the first two PCs that accounted for around 88% of the total variance (Figure 5E). The first axis differentiated some cursorial species and some terrestrial species with positive scores from the remaining taxa (Figure 5E, *x* axis). The second PC mainly differentiated the semifossorial European badger plus some cursorial and terrestrial species with positive scores from other taxa (Figure 5E, *y* axis). The morphological changes associated with these eigenvectors are shown in Figure 5F and Additional file 4C.

## Discussion

### Phylogeny and allometry are significant sources of bone variation

The permutation test showed that phylogeny influences the shape and size of the hind limb bones (Table 1). These results are in line with those obtained for the forelimb bones [13,26,28,38]. It appears that the shape and size of the carnivoran appendicular skeleton were acquired early during the evolution of each family and were maintained with little variation during their subsequent evolution.

The shape of the hindlimb is strongly influenced by size differences (i.e., allometry) and this association is not merely due to a phylogenetic correlation (Figures 1 and 2). Given the similarity between these results and previous findings for the forelimb bones [38], we suggest that the shape of the entire appendicular skeleton of fissiped carnivorans is strongly influenced by size differences. These results are in line with previous research on limb-bone scaling in mammals [18,21,44].

With the sole exception of the tibia, the allometric changes were related to bone robustness (Figures 1B, 1D and 2B, and 2D) and probably indicate the need of larger animals to manage increasing stresses due to their large body size [44]. However, increased bone robustness is not the only way to reduce peak stresses in large-sized animals; the adoption of a more upright posture also reduces bending stresses and increases the effective mechanical advantage of muscles [20,45]. The size-related shape changes shown for the tibia involve an increase of shaft curvature and a change in the condyles in the proximal epiphysis to a more horizontal position (Figures 1F and 2F). These shape changes could be related to large-sized species needing to adopt a more upright posture because these changes enhance resistance to axial stresses at the expense of bending stresses [20].

The allometric changes in the hind limb bones described above are generally equivalent to those obtained for the forelimb bones shape described in Martín-Serra *et al.*[38].

### Morphological variability and phylomorphospaces

The difference in phylogenetic conservatism between the PCA obtained for the pelvis and the PCAs obtained for the long bones are equivalent to findings obtained for the forelimb [38]. We found that the scapula was a more conservative bone than the humerus or the radius-ulna complex. This implies that the tight connection of the pelvis to the axial skeleton is not a potential cause of its phylogenetic conservatism. This is because the scapula is not directly connected to the axial skeleton and is also a highly conservative bone. Similarly, the fact that the scapula is composed mainly by a single element (as the coracoid is a small process with little relevance compared with the main body of the scapula [46]) could indicate that the more complex structure of the pelvis (formed by three different fused elements) is not a feasible explanation for its phylogenetic conservatism. In contrast, the differences between the proximal and distal elements of the limbs could be explained by their different developmental origin [47].

The first PC of the size-free shapes of the femur and tibia shows that the main axis of shape variation is a gradient of slenderness-robustness (Figure 3C and 3F). However, there are numerous morphological similarities among distantly related taxa with different ecologies. On the one hand, having slender bones is common to most canine canids, hyenids, the extinct “dog-like” bear *Hemicyon*, the cheetah, the bobcat, and the serval. However, having slender bones is a morphological solution, which could be favoured by natural selection for different purposes such as the active pursuit of prey (e.g., the cheetah), long-distance pursuit (e.g., wolves), or long-distance foraging (e.g., foxes). In any case, slender hind limb bones indicate cursorial adaptations, i.e., an increased capacity to run faster and/or to run for longer distances with more energetic efficiency [5,9,24,48-50]. On the other hand, distantly related taxa with different ecologies also share extremely robust hind limb bones. For example, the European badger, the extinct cave bear, and some procyonids share robust femora and tibiae. This is also the case for the extinct *Patriofelis* (order Creodonta), the false saber-toothed cats *Barbourofelis* and *Hoplophoneus*, and the saber-tooth *Smilodon*. In mammalian carnivores, having robust limb bones is thought to be an adaptation in order to resist axial and bending stresses [29] related to multiple activities such as moving excavated soil during digging (e.g., the European badger) or withstanding body weight loads generated during hunting in large cats [25,29,33,36,51,52].

In summary, several distantly related taxa adapted to different ecological habits and functional necessities share bone morphologies that involve having slender or robust limbs. We found little differentiation between the behavioural categories, which could be due to the fact that many ecological contexts could favour one solution or another.

### Locomotor behaviour is only partially reflected by the shape of the hind limb bones

The six ecological groups were not clearly differentiated by the between-group PCAs performed to investigate the effects of ecology on bone shape variation. Species are differentiated according to their phylogenetic relationships. For example, bone morphology does not differentiate terrestrial canids from cursorial canids and terrestrial felids are not differentiated from scansorial felids. A visual inspection of the phylomorphospaces shows a clear phylogenetic effect in the distribution of taxa because internal branches are larger than more terminal branches (Figure 5A, 5C and 5E). These results were expected, as other authors have found that phylogeny strongly influences bone morphology and locomotor behaviour [27,53].

## Conclusions

This article has demonstrated that the shape of the hind limb bones is strongly influenced by size differences. In addition, allometric shape changes show that large-sized species have pelvises with larger areas for the attachment of proximal limb muscles. They also have more robust femora. The shape of their tibiae suggests that they have a more upright posture compared to smaller species. These allometric shape changes are not merely due to a phylogenetic pattern. Nevertheless, phylogeny and size have a strong influence on limb bone shape. Furthermore, the phenotypic spaces indicated that, once size effects are discarded, the main axis of shape variation is still a gradient of slenderness-robustness. We hypothesized that this axis reflects an adaptive trade-off between maintaining energetic efficiency during locomotion – acquired by having slender bones – and resisting high peak stresses – acquired by having robust bones. However, both morphological extremes can be adaptive in multiple ecological scenarios and behavioural contexts leading to a lack of a one-to-one correspondence between morphology and function. Thus, several species with very different ecologies have similar hind limb bone shapes, which is probably due to the presence of strong biomechanical and phylogenetic constraints that mask the association between locomotor behaviour and bone shape. In fact, we found that the ecological influence on limb bone shape was very weak when we analysed specific morphological differences between several ecological groups.

The pattern of hind limb shape evolution described in this article is equivalent to the pattern of forelimb shape evolution [38]. This tight correspondence between the fore- and hind limb in shape evolution means that future studies can investigate the patterns of morphological integration between both limbs from structural and functional perspectives. Thus, we suggest that the entire appendicular skeleton of mammalian carnivores represents a conspicuous example of a “one-to-many” pattern of evolution between phenotype and function. Strikingly, this pattern of evolution is in complete contrast to the “many-to-one” pattern for the evolution of the craniodental skeleton in which similar morphological solutions evolved multiple times in different lineages to accomplish similar functions such as feeding [54]. This suggests that the appendicular skeleton could be more constrained than the crania, probably because of the strong biomechanical constraints imposed by active locomotion.

## Methods

### Data

The data set included 135 pelvises, 194 femora, and 194 tibiae from 46 species of modern carnivorans and 27 extinct ones (see Additional file 3: Tables S1, S2 and S3). Modern and extinct species were selected to include the highest morphological variability within each family as far as possible. We also included *Patriofelis*† or *Hyaenodon pervagus*† (Mammalia, Creodonta) whenever possible with a similar purpose, i.e., to increase morphological variability by including an example of a closely related mammalian order, such as Creodonta [55]. Adult specimens alone were included to avoid the effects of ontogenetic variation. Adults were defined by the complete fusion of the epiphysis to the diaphysis. All the specimens were housed in the following institutions: American Museum of Natural History (AMNH, New York), Natural History Museum (NHM, London), Naturhistorisches Museum (NMB, Basel), Museo Nacional de Ciencias Naturales (MNCN, Madrid), Museo di Storia Naturale (MSN, Firenze), Staten Naturhistoriske Museum (SNM, Copenhagen), and Museo de Ciencias Naturales de Valencia (MCNV, Valencia).

### Digitized landmarks and three-dimensional model construction

**Figure 6:**
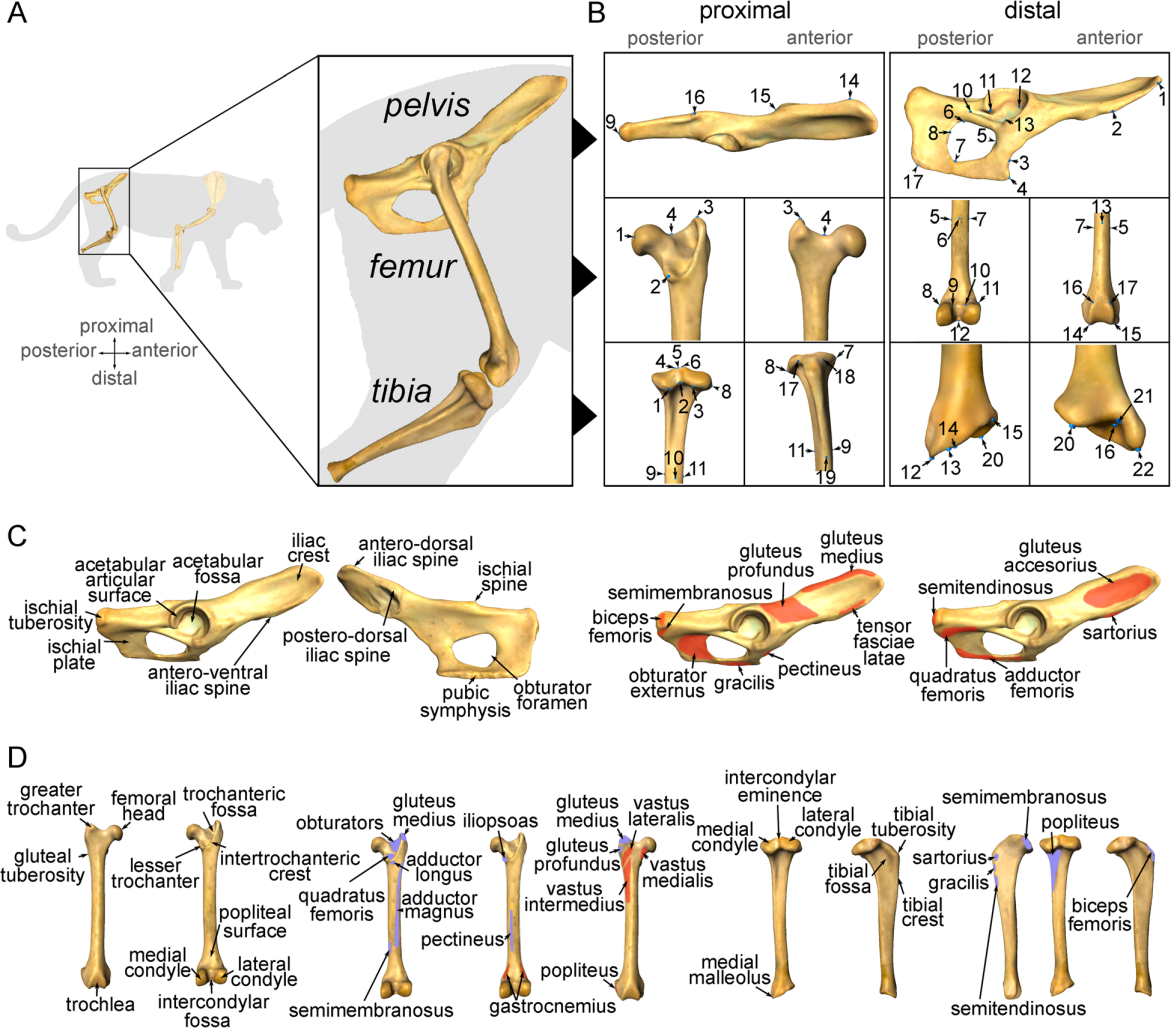
**A three-dimensional analysis of hind limb evolution in carnivores. A**, main bones of the hind limb analysed in this paper. **B**, Landmarks used in the morphometric analyses of the hind limb bones (for detailed descriptions see Additional file 3: Table S4). **C**, key morphological features in the carnivoran pelvis of. **D**, main morphological structures in the femur and tibia of carnivorans. The muscle origins (red) and insertions (purple) of the main muscles involved in locomotion are also shown for each hind limb bone. (Anatomical keys were obtained from Barone [56] and Homberger and Walker [57]).

A set of three-dimensional landmarks was digitized using a Microscribe G2X. Their 3D coordinates (*x*,*y*,*z*) were imported into Exce using the Immersion software package (Immersion, Inc., San Jose, CA, USA). These landmarks (Figure 6) were chosen to capture the most important morphological aspects of the hind limb bones [56,57] (Additional file 3: Table S4).

Shape visualizations at the extremes of the multivariate axes were performed by warping the scanned surface of a *Panthera onca* (femur and tibia) and an *Uncia uncia* (pelvis), using Landmark software [58] (see [38] for further details).

We performed a Procrustes fit [59] using the landmark coordinates. To avoid the effects of static allometry, we averaged the Procrustes coordinates (Pco) and Centroid size (Cs) by species. We averaged by genus those specimens not identified at the species level (e.g., *Tomarctus* sp., *Hoplophoneus* sp.). Only in the case of *Smilodon* sp. we averaged by genus in order to avoid taxonomical uncertainty at the genus level. These procedures and all the following statistical analyses were performed using the MorphoJ software [60]. All these morphometric data are available in Additional file 5.

### The phylogenetic signal in limb bone shape and size

**Figure 7:**
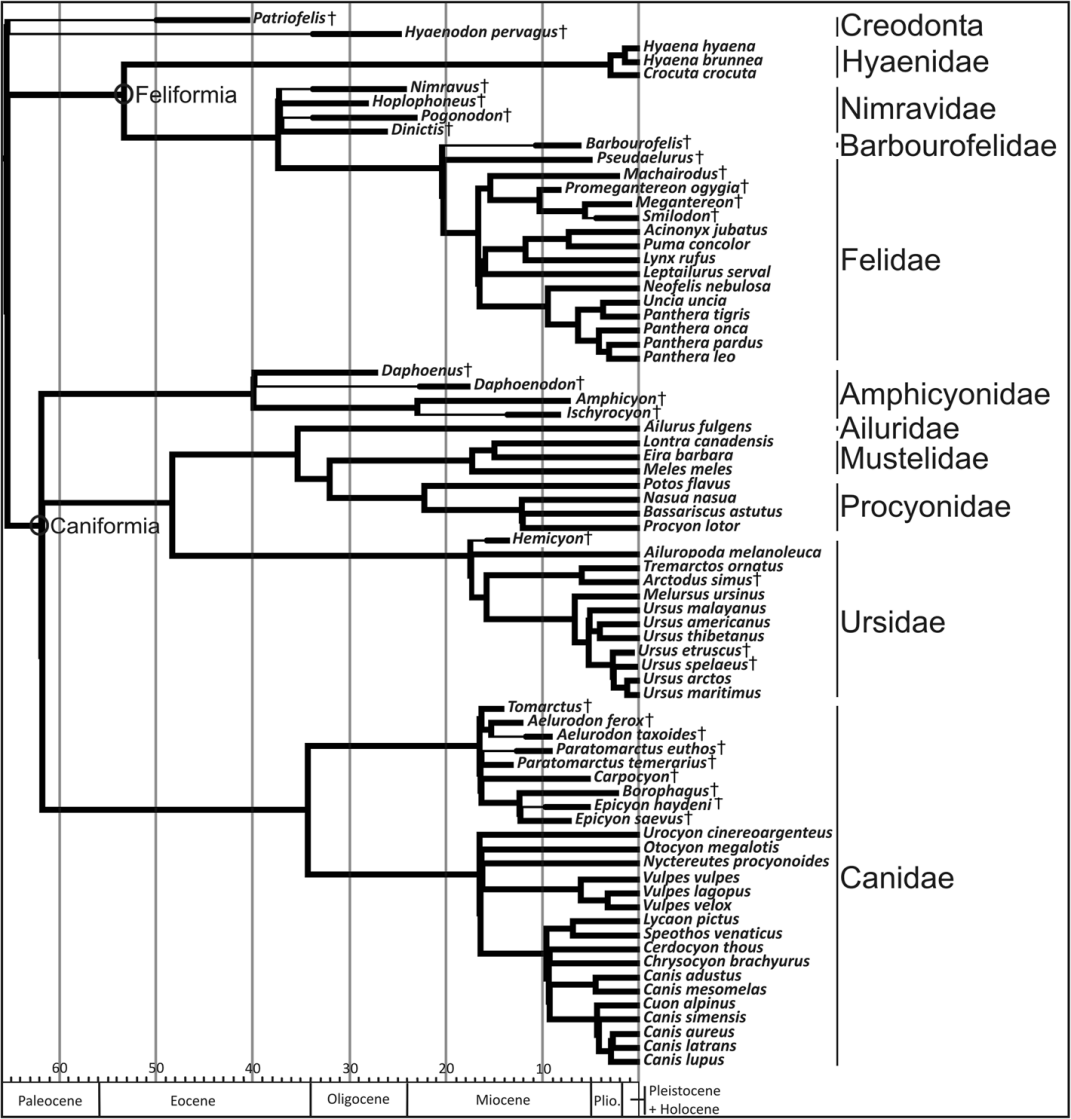
**Phylogenetic tree topology of carnivoran species used in this study.** The extinct creodonts (order Creodonta) *Patriofelis* sp. and *Hyaenodon pervagus* are used as outgroups to root the tree (see text for details). Tree topology and branch lengths were taken from the literature (Additional file 3: Table S5).

Mesquite software [61] was used to construct a phylogenetic tree (Figure 7) to assess phylogenetic patterns in the sample following information in published sources. Tree topology was constructed using the trees published by Nyakatura and Bininda-Emonds [37] and Koepfli *et al.*[62] (Additional file 6). The phylogenetic relationships of extinct species were assigned following information in published sources (Additional file 3: Table S5). We included branch lengths in million years before present in our composite phylogeny [63-65]. Information on the time of divergence between living taxa was obtained from Nyakatura and Bininda-Emonds [37] and Koepfli *et al*. [62]. The branch lengths of extinct species were inferred from the stratigraphic range of taxa from different references and public databases (Additional file 3: Table S5).

A permutation test was used to assess the presence of a phylogenetic signal in bone shape and size [66,67] (see [68] for more details).

### The effect of size on limb bone shape

Multivariate regression [69] was performed to evaluate the effects of interspecific allometry of shape (Pco) on size (Log-transformed Cs) for each bone. However, species cannot be treated as statistically independent data points because they are related by phylogeny [70]. Thus, independent contrasts analysis [71] was applied to the shape and size of limb bones. Multivariate regression of independent contrasts for shape on independent contrasts for Log-transformed centroid size was performed to investigate the effects of evolutionary allometry. Finally, these regression vectors were applied to the species dataset to obtain the residuals following the method of Klingenberg and Marugán-Lobón [72]. These residuals were used in all multivariate analyses.

A permutation test (10,000 iterations) was used to assess the statistical significance of all the regressions versus the null hypothesis of complete size independence [73].

### Phenotypic variability and evolution

Principal Components Analysis was used to investigate phenotypic variation from the covariance matrix of the shape of the bones. In addition, to reconstruct the phylogenetic history of phenotypic space occupation, we created phylomorphospaces for each hind limb bone [38,64,67,68,74-79].

### The influence of locomotor behaviour on limb bone shape

We classified extant carnivoran species within different locomotor groups (Additional file 3: Table S1) following the categories of Samuels *et al*. [27] to quantify the influence of locomotor behaviour on limb bone shape: *(1)* cursorial, i.e., species that display rapid locomotion on the ground by galloping; *(2)* scansorial, i.e., species that are able of climbing but do not forage in trees; *(3)* arboreal, i.e., species that forage in trees; *(4)* semifossorial, i.e., species that typically dig; *(5)* semiaquatic, i.e., species that typically swim; and *(6)* terrestrial, i.e., species that do not climb, swim, or typically run quickly.

A between-group PCA was performed following the approach of Mitteroecker and Bookstein [80]. We averaged the size-free shapes of all the species within the six locomotor groups. We then computed the PCs from these six averages and plotted the species by applying these eigenvectors to the species. This methodology reveals the morphological axes that better differentiate the group averages. In addition, between-group PCA avoids the problems of Canonical Variate Analysis associated with a small within-group sample size when the dimensionality of the data is high [80]. Subsequently, we created between-group phylomorphospaces for each limb bone shape. We used these phylomorphospaces to investigate whether there is a phylogenetic pattern in the distribution of species even though the PCs were obtained to specifically investigate ecological influences in shape variation.

## Availability of supporting data

The data sets supporting the results of this article are available in the Dryad Digital Repository: doi:10.5061/dryad.8h6nf [81].

## Competing interests

The authors declare that they have no competing interests.

## Authors’ contributions

AMS collected the data. AMS and BF conducted the analyses. AMS, BF, and PP wrote the manuscript. PP provided advice on the analyses. AMS, BF, and PP conceived and designed the study. All the authors read and approved the final manuscript.

## Supplementary Material

Additional file 1 **Interactive three-dimensional models of shape variation in the carnivoran hind limb.** Size-related shape changes for pelvis **(A)**, femur **(B)** and tibia **(C)**. Left indicates negative regression scores and right positive scores. Click here for file

Additional file 2 **Three-dimensional models showing the shape changes obtained from the PCAs.** Pelvis **(A)**, femur **(B)** and tibia **(C)**. PC I top, PC II bottom; left for negative scores, right for positive scores. Click here for file

Additional file 3 Supporting tables and references. Click here for file

Additional file 4 **Three-dimensional models showing the shape changes obtained from the between-group PCAs.** Pelvis **(A)**, femur **(B)** and tibia **(C)**. PC I top, PC II bottom; left for negative scores, right for positive scores. Click here for file

Additional file 5 **Table in Excel format with morphometric data averaged per species for each hind limb bone analysed.** Centroid size, log-transformed centroid size and Procrustes coordinates are shown for pelvis (1^st^ and 2^nd^ sheets), femur (3^rd^ and 4^th^ sheets) and tibia (5^th^ and 6^th^ sheets). 1^st^, 3^rd^ and 5^th^ sheets show values for all the species; 2^nd^, 4^th^ and 6^th^ sheets include only extant species. Click here for file

Additional file 6 Nexus file of the composite tree used in this paper. Click here for file
