# Supplementary figures and images for: A three-dimensional analysis of the morphological evolution and locomotor behaviour of the carnivoran hind limb
Source: BMC Evol Biol. 2014 Jun 14;14:129. doi: 10.1186/1471-2148-14-129 (PMC4065579; doi:10.1186/1471-2148-14-129)

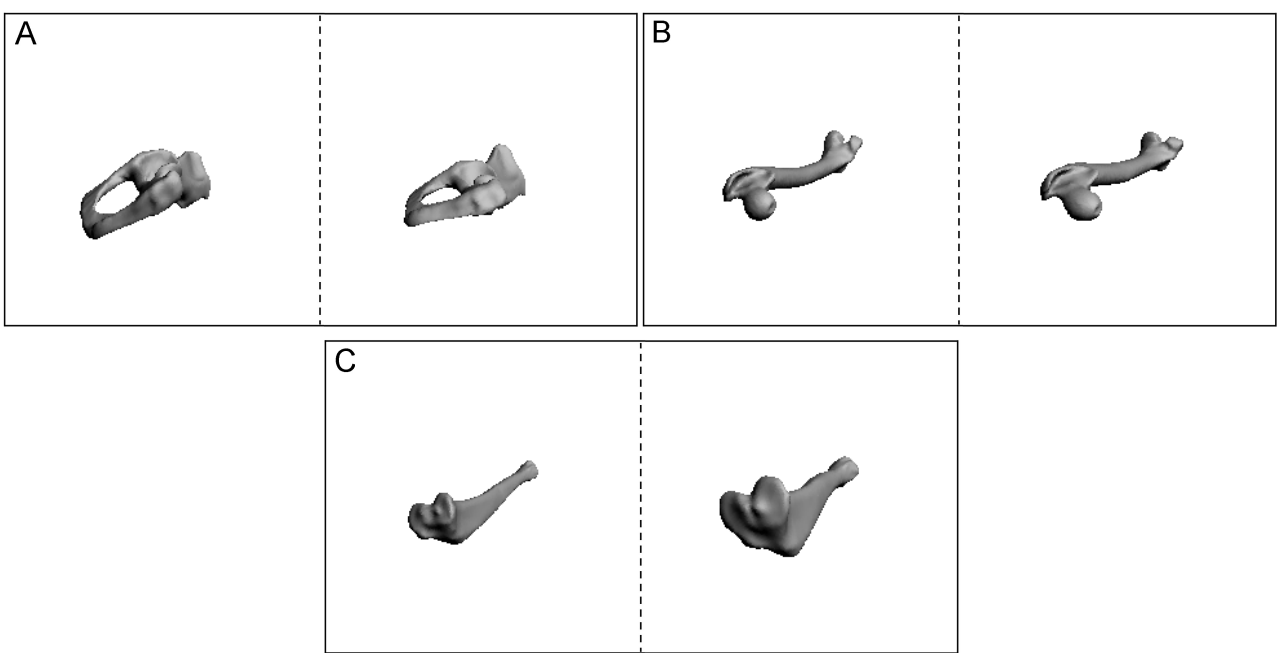

Supplement: Additional file 1 — Interactive three-dimensional models of shape variation in the carnivoran hind limb. Size-related shape changes for pelvis (A), femur (B) and tibia (C). Left indicates negative regression scores and right positive scores. [file 1471-2148-14-129-S1.pdf]

A

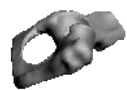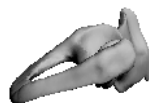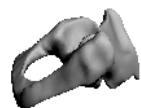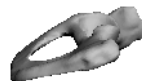

B

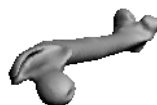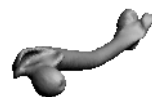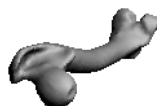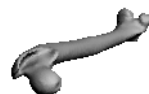

C

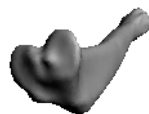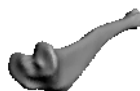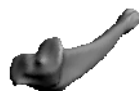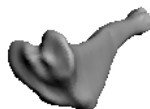

Supplement: Additional file 2 — Three-dimensional models showing the shape changes obtained from the PCAs. Pelvis (A), femur (B) and tibia (C). PC I top, PC II bottom; left for negative scores, right for positive scores. [file 1471-2148-14-129-S2.pdf]

A

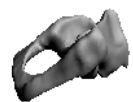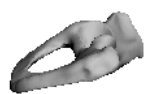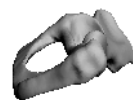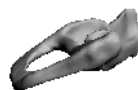

B

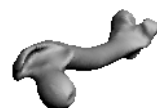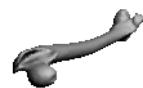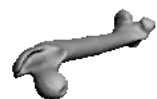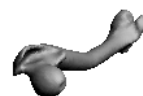

C

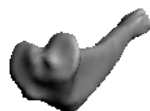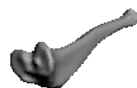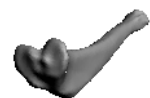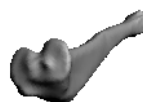

Supplement: Additional file 4 — Three-dimensional models showing the shape changes obtained from the between-group PCAs. Pelvis (A), femur (B) and tibia (C). PC I top, PC II bottom; left for negative scores, right for positive scores. [file 1471-2148-14-129-S4.pdf]
