# Supplementary material for: A three-dimensional analysis of the morphological evolution and locomotor behaviour of the carnivoran hind limb
Source: BMC Evol Biol. 2014 Jun 14;14:129. doi: 10.1186/1471-2148-14-129 (PMC4065579; doi:10.1186/1471-2148-14-129)
Supplement: Additional file 3 — Supporting tables and references. [file 1471-2148-14-129-S3.doc]

**Additional file 3**

Table S1. Sample sizes used in this study for each forelimb bone (P, pelvis; F, femur; T, tibia). Locomotor category established for each species according to Samuels *et al*. (2013) [1] are shown. Daggers (†) represents extinct species.

| **Family** | **Species (abbreviation)** | **P** | **F** | **T** | **Locomotor category** |
| --- | --- | --- | --- | --- | --- |
| **Ailuridae** | *Ailurus fulgens (Afu)* | 2 | 2 | 2 | Arboreal |
| **Amphicyonidae** | *Amphicyon* sp. *(Amp)* † | 1 | 6 | 11 | - |
|  | *Daphoenodon* sp. *(Dad)* † | 0 | 0 | 1 | - |
|  | *Daphoenus* sp. *(Dan)* † | 0 | 1 | 1 | - |
|  | *Ischyrocyon* sp. *(Isc)* † | 0 | 2 | 2 | - |
| **Barbourofelidae** | *Barbourofelis* sp. *(Bar)* † | 0 | 1 | 2 | - |
| **Canidae** | *Aelurodon ferox (Afe)* † | 0 | 3 | 2 | - |
|  | *Aelurodon taxoides (Ata)* † | 0 | 2 | 0 | - |
|  | *Borophagus* sp. *(Bor)* † | 0 | 0 | 4 | - |
|  | *Canis adustus (Cad)* | 1 | 1 | 1 | Cursorial |
|  | *Canis aureus (Cau)* | 2 | 2 | 2 | Cursorial |
|  | *Canis latrans (Cla)* | 5 | 5 | 5 | Cursorial |
|  | *Canis lupus (Clu)* | 5 | 5 | 5 | Cursorial |
|  | *Canis mesomelas (Cme)* | 4 | 4 | 4 | Cursorial |
|  | *Canis simensis (Csi)* | 1 | 1 | 1 | Cursorial |
|  | *Carpocyon* sp. *(Car)* † | 0 | 2 | 0 | - |
|  | *Cerdocyon thous (Cth)* | 5 | 5 | 5 | Terrestrial |
|  | *Chrysocyon brachyurus (Cbr)* | 2 | 2 | 2 | Terrestrial |
|  | *Cuon alpinus (Cal)* | 4 | 4 | 4 | Cursorial |
|  | *Epicyon haydeni (Eha)* † | 0 | 1 | 4 | - |
|  | *Epicyon saevus (Esa)* † | 0 | 2 | 1 | - |
|  | *Lycaon pictus (Lpi)* | 2 | 2 | 2 | Cursorial |
|  | *Nyctereutes procyonoides (Npr)* | 2 | 2 | 2 | Terrestrial |
|  | *Otocyon megalotis (Ome)* | 2 | 2 | 2 | Cursorial |
|  | *Paratomarctus euthos (Peu)* † | 0 | 0 | 2 | - |
|  | *Paratomarctus temerarius (Pte)* † | 0 | 2 | 0 | - |
|  | *Speothos venaticus (Sve)* | 2 | 2 | 1 | Terrestrial |
|  | *Tomarctus* sp. *(Tom)* † | 0 | 6 | 5 | - |
|  | *Urocyon cinereoargenteus (Uci)* | 4 | 4 | 4 | Scansorial |
|  | *Vulpes lagopus (Vla)* | 2 | 2 | 2 | Terrestrial |
|  | *Vulpes velox (Vve)* | 2 | 2 | 2 | Cursorial |
|  | *Vulpes vulpes (Vvu)* | 3 | 3 | 3 | Cursorial |
| **Creodonta** | *Hyaenodon pervagus (Hpe)* † | 0 | 0 | 1 | - |
|  | *Patriofelis* sp. *(Pat)* † | 0 | 1 | 0 | - |
| **Felidae** | *Acinonyx jubatus (Aju)* | 5 | 5 | 5 | Cursorial |
|  | *Leptailurus serval (Lse)* | 1 | 2 | 2 | Terrestrial |
|  | *Lynx rufus (Lru)* | 4 | 4 | 4 | Scansorial |
|  | *Machairodus* sp. *(Mac)* † | 1 | 2 | 3 | - |
|  | *Megantereon* sp. *(Meg)* † | 0 | 1 | 1 | - |
|  | *Neofelis nebulosa (Nne)* | 1 | 1 | 1 | Arboreal |
|  | *Panthera leo (Ple)* | 5 | 5 | 5 | Terrestrial |
|  | *Panthera onca (Pon)* | 3 | 4 | 4 | Scansorial |
|  | *Panthera pardus (Ppa)* | 6 | 6 | 6 | Scansorial |
|  | *Panthera tigris (Pti)* | 4 | 4 | 4 | Terrestrial |
|  | *Promegantereon ogygia (Pro)* † | 1 | 4 | 2 | - |
|  | *Pseudaelurus* sp. *(Pse)* † | 1 | 1 | 2 | - |
|  | *Puma concolor (Pco)* | 4 | 4 | 4 | Scansorial |
|  | *Smilodon* sp. *(Smi)* † | 1 | 4 | 4 | - |
|  | *Uncia uncia (Uun)* | 4 | 4 | 4 | Scansorial |
| **Hyaenidae** | *Crocuta crocuta (Ccr)* | 5 | 5 | 5 | Cursorial |
|  | *Hyaena brunnea (Hbr)* | 1 | 1 | 1 | Cursorial |
|  | *Hyaena hyaena (Hhy)* | 2 | 2 | 2 | Cursorial |
| **Mustelidae** | *Eira barbara (Eba)* | 2 | 2 | 2 | Arboreal |
|  | *Lontra canadensis (Lca)* | 2 | 2 | 2 | Semiaquatic |
|  | *Meles Meles (Mme)* | 1 | 1 | 1 | Semifossorial |
| **Nimravidae** | *Dinictis* sp. *(Din)* † | 0 | 5 | 0 | - |
|  | *Hoplophoneus* sp. *(Hop)* † | 1 | 4 | 1 | - |
|  | *Nimravus* sp. *(Nim)* † | 0 | 1 | 0 | - |
|  | *Pogonodon* sp. *(Pog)* † | 0 | 1 | 2 | - |
| **Procyonidae** | *Bassariscus astutus (Bas)* | 1 | 1 | 1 | Arboreal |
|  | *Nasua nasua (Nna)* | 1 | 1 | 1 | Scansorial |
|  | *Potos flavus (Pfl)* | 3 | 3 | 3 | Arboreal |
|  | *Procyon lotor (Plo)* | 3 | 3 | 3 | Scansorial |
| **Ursidae** | *Ailuropoda melanoleuca (Ame)* | 4 | 4 | 4 | Terrestrial |
|  | *Arctodus simus (Asi)* † | 1 | 3 | 4 | - |
|  | *Helarctos malayanus (Hma)* | 1 | 1 | 1 | Scansorial |
|  | *Hemicyon* sp. *(Hem)* † | 0 | 1 | 1 | - |
|  | *Melursus ursinus (Mur)* | 3 | 3 | 3 | Terrestrial |
|  | *Tremarctos ornatos (Tor)* | 1 | 1 | 1 | Scansorial |
|  | *Ursus americanus (Uam)* | 3 | 3 | 3 | Scansorial |
|  | *Ursus arctos (Uar)* | 4 | 4 | 4 | Terrestrial |
|  | *Ursus etruscus (Uet)* † | 0 | 0 | 2 | - |
|  | *Ursus maritimus (Uma)* | 2 | 4 | 4 | Semiaquatic |
|  | *Ursus spelaeus (Usp)* † | 0 | 2 | 3 | - |
|  | *Ursus thibetanus (Uth)* | 3 | 3 | 3 | Scansorial |

Table S2. List of specimens for the living species included in this paper. Host institution and identity number (ID) are indicated. AMNH, American Museum of Natural History (New York); NHM, Natural History Museum (London). * Indicates a specimen in which the pelvis (P) or the tibia (T) was absent; the three bones analyzed here were present for the remaining specimens.

| **Species** | **ID** | **Host Institution** |
| --- | --- | --- |
| *Acinonyx jubatus* | 119654 | AMNH |
| *Acinonyx jubatus* | 119655 | AMNH |
| *Acinonyx jubatus* | 119656 | AMNH |
| *Acinonyx jubatus* | 1940.1.20.17 | NHM |
| *Acinonyx jubatus* | 1962.7.6.15 | NHM |
| *Ailuropoda melanoleuca* | 147746 | AMNH |
| *Ailuropoda melanoleuca* | 110454 | AMNH |
| *Ailuropoda melanoleuca* | 89028 | AMNH |
| *Ailuropoda melanoleuca* | 89030 | AMNH |
| *Ailurus fulgens* | 185346 | AMNH |
| *Ailurus fulgens* | 80164 | AMNH |
| *Bassariscus astutus* | 182560 | AMNH |
| *Canis adustus* | 114174 | AMNH |
| *Canis aureus* | 187714 | AMNH |
| *Canis aureus* | 54516 | AMNH |
| *Canis latrans* | 123183 | AMNH |
| *Canis latrans* | 1317 | AMNH |
| *Canis latrans* | 1316 | AMNH |
| *Canis latrans* | 141170 | AMNH |
| *Canis latrans* | 141153 | AMNH |
| *Canis lupus* | 98226 | AMNH |
| *Canis lupus* | 98227 | AMNH |
| *Canis lupus* | 98225 | AMNH |
| *Canis lupus* | 134941 | AMNH |
| *Canis lupus* | 134942 | AMNH |
| *Canis mesomelas* | 34734 | AMNH |
| *Canis mesomelas* | 187712 | AMNH |
| *Canis mesomelas* | 187713 | AMNH |
| *Canis mesomelas* | 114228 | AMNH |
| *Canis simensis* | 81001 | AMNH |
| *Cerdocyon thous* | 134049 | AMNH |
| *Cerdocyon thous* | 214709 | AMNH |
| *Cerdocyon thous* | 214703 | AMNH |
| *Cerdocyon thous* | 209123 | AMNH |
| *Cerdocyon thous* | 209128 | AMNH |
| *Chrysocyon brachyurus* | 133941 | AMNH |
| *Chrysocyon brachyurus* | 133940 | AMNH |
| *Crocuta crocuta* | 35358 | AMNH |
| *Crocuta crocuta* | 83593 | AMNH |
| *Crocuta crocuta* | 52097 | AMNH |
| *Crocuta crocuta* | 187769 | AMNH |
| *Crocuta crocuta* | 187776 | AMNH |
| *Cuon alpinus* | 102083 | AMNH |
| *Cuon alpinus* | 54976 | AMNH |
| *Cuon alpinus* | 54984 | AMNH |
| *Cuon alpinus* | 54842 | AMNH |
| *Eira barbara* | 214736 | AMNH |
| *Eira barbara* | 23487 | AMNH |
| *Helarctos malayanus* | 35364 | AMNH |
| *Hyaena brunnea* | 1962-7.23.1 | NHM |
| *Hyaena hyaena* | 244436 | AMNH |
| *Hyaena hyaena* | 54512 | AMNH |
| *Leptailurus serval* (P)* | 119207 | AMNH |
| *Leptailurus serval* | 27837 | AMNH |
| *Lutra canadensis* | 165762 | AMNH |
| *Lutra canadensis* | 182561 | AMNH |
| *Lycaon pictus* | 82085 | AMNH |
| *Lycaon pictus* | 85154 | AMNH |
| *Lynx rufus* | 119206 | AMNH |
| *Lynx rufus* | 208417 | AMNH |
| *Lynx rufus* | 128527 | AMNH |
| *Lynx rufus* | 171361 | AMNH |
| *Meles meles* | 70604 | AMNH |
| *Melursus ursinus* | 150205 | AMNH |
| *Melursus ursinus* | 54465 | AMNH |
| *Melursus ursinus* | 54464 | AMNH |
| *Nasua nasua* | 214722 | AMNH |
| *Neofelis nebulosa* | 238650 | AMNH |
| *Nyctereutes procyonoides* | 249766 | AMNH |
| *Nyctereutes procyonoides* | 249767 | AMNH |
| *Otocyon megalotis* | 233011 | AMNH |
| *Otocyon megalotis* | 63993 | AMNH |
| *Panthera leo* | 1952.10.20.13 | NHM |
| *Panthera leo* | 112.a | NHM |
| *Panthera leo* | 1857.2.24.1 | NHM |
| *Panthera leo* | 75.1998 | NHM |
| *Panthera leo* | 75.945 | NHM |
| *Panthera onca* | 35571 | AMNH |
| *Panthera onca* | 139959 | AMNH |
| *Panthera onca* (P)* | 135928 | AMNH |
| *Panthera onca* | 1858.5.26.9 | NHM |
| *Panthera pardus* | 209087 | AMNH |
| *Panthera pardus* | 1940.1.20.18 | NHM |
| *Panthera pardus* | 115p | NHM |
| *Panthera pardus* | 1851.2.17.3 | NHM |
| *Panthera pardus* | 1940.1.20.20 | NHM |
| *Panthera pardus* | 1849.6.20.2 | NHM |
| *Panthera tigris* | 113743 | AMNH |
| *Panthera tigris* | 113748 | AMNH |
| *Panthera tigris* | 135846 | AMNH |
| *Panthera tigris* | 85404 | AMNH |
| *Potos flavus* | 266597 | AMNH |
| *Potos flavus* | 265959 | AMNH |
| *Potos flavus* | 266599 | AMNH |
| *Procyon lotor* | 173897 | AMNH |
| *Procyon lotor* | 147436 | AMNH |
| *Procyon lotor* | 237438 | AMNH |
| *Puma concolor* | 1335 | AMNH |
| *Puma concolor* | 90213 | AMNH |
| *Puma concolor* | 14026 | AMNH |
| *Puma concolor* | 135341 | AMNH |
| *Speothos venaticus* | 52.1086 | NHM |
| *Speothos venaticus* (T)* | 1966.1.24.1 | NHM |
| *Tremarctos ornatus* | 81.784 | NHM |
| *Uncia uncia* | 207704 | AMNH |
| *Uncia uncia* | 266952 | AMNH |
| *Uncia uncia* | 119662 | AMNH |
| *Uncia uncia* | 100110 | AMNH |
| *Urocyon cinereoargenteus* | 35695 | AMNH |
| *Urocyon cinereoargenteus* | 90134 | AMNH |
| *Urocyon cinereoargenteus* | 148799 | AMNH |
| *Urocyon cinereoargenteus* | 137028 | AMNH |
| *Ursus americanus* | 128521 | AMNH |
| *Ursus americanus* | 98950 | AMNH |
| *Ursus americanus* | 45149 | AMNH |
| *Ursus arctos* | 14054 | AMNH |
| *Ursus arctos* | 135502 | AMNH |
| *Ursus arctos* | 45150 | AMNH |
| *Ursus arctos* | 70254 | AMNH |
| *Ursus maritimus* | 35065 | AMNH |
| *Ursus maritimus* | 31573 | AMNH |
| *Ursus maritimus* (P)* | 215283 | AMNH |
| *Ursus maritimus* (P)* | 75244 | AMNH |
| *Ursus thibetanus* | 70320 | AMNH |
| *Ursus thibetanus* | 80248 | AMNH |
| *Ursus thibetanus* | 23086 | AMNH |
| *Vulpes lagopus* | 28117 | AMNH |
| *Vulpes lagopus* | 28116 | AMNH |
| *Vulpes velox* | 35392 | AMNH |
| *Vulpes velox* | 100215 | AMNH |
| *Vulpes vulpes* | 69550 | AMNH |
| *Vulpes vulpes* | 128487 | AMNH |
| *Vulpes vulpes* | 128486 | AMNH |

Table S3. List of fossil specimens included in this paper. Host institution and identity number (ID) are indicated. AMNH, American Museum of Natural History (New York); NHM, Natural History Museum (London); NMB, Naturhistorisches Museum (Basel); MNCN, Museo Nacional de Ciencias Naturales (Madrid); MSN, Museo di Storia Naturale (Firenze); SNM, Staten Naturhistoriske Museum (Copenhagen); MCNV, Museo de Ciencias Naturales de Valencia (Valencia).

| **Species** | **ID** | **Host Institution** |
| --- | --- | --- |
| **Pelvis** |  |  |
| *Amphicyon* sp. | Cast | AMNH |
| *Arctodus simus* | 12392 | AMNH |
| *Hoplophoneus primaevus* | 38980 | AMNH |
| *Machairodus aphanistus* | BAT-1-'06-F6-58 | MNCN |
| *Promegantereon ogygia* | B-466 | MNCN |
| *Pseudaelurus* sp. | 62210-B | AMNH |
| *Smilodon fatalis* | LB41 | NMB |
| **Femur** |  |  |
| *Aelurodon ferox* | 27479 L | AMNH |
| *Aelurodon ferox* | 27479 R | AMNH |
| *Aelurodon ferox* | 67467 | AMNH |
| *Aelurodon taxoides* | 67446 | AMNH |
| *Aelurodon taxoides* | 67447 | AMNH |
| *Amphicyon* sp. | Cast | AMNH |
| *Amphicyon ingens* | 68117 | AMNH |
| *Amphicyon ingens* | 68147 | AMNH |
| *Amphicyon* sp. | 68100-A | AMNH |
| *Amphicyon* sp. | 68104-A | AMNH |
| *Amphicyon* sp. | 68111-C | AMNH |
| *Arctodus simus* | 12392 L | AMNH |
| *Arctodus simus* | 12392 R | AMNH |
| *Arctodus simus* | 25531 | AMNH |
| *Barbourofelis fricki* | 61986 | AMNH |
| *Carpocyon tagarctus* | 67559 | AMNH |
| *Carpocyon tagarctus* | 67560 | AMNH |
| *Daphoenus minimus* | 63343 | AMNH |
| *Dinictis* sp. | 125652 | AMNH |
| *Dinictis* sp. | 62074 | AMNH |
| *Dinictis* sp. | 62122 | AMNH |
| *Dinictis* sp. | 69425 | AMNH |
| *Dinictis* sp. | 69426 | AMNH |
| *Epicyon haydeni* | 67613 | AMNH |
| *Epicyon saevus* | 67505 | AMNH |
| *Epicyon saevus* | 8305 | AMNH |
| *Hemicyon* sp. | 68176 | AMNH |
| *Hoplophoneus primaevus* | 38980 | AMNH |
| *Hoplophoneus* sp. | 38981 | AMNH |
| *Hoplophoneus* sp. | 62077 | AMNH |
| *Hoplophoneus* sp. | 62090 | AMNH |
| *Ischyrocyon* sp. | 68153-A | AMNH |
| *Ischyrocyon* sp. | 68153 | AMNH |
| *Machairodus aphanistus* | B-199 | MNCN |
| *Machairodus* sp. | 104727 | AMNH |
| *Megantereon cultridens* | Se311 | NMB |
| *Nimravus brachyops* | 6935 | AMNH |
| *Paratomarctus temerarius* | 105338 | AMNH |
| *Paratomarctus temerarius* | 61071 | AMNH |
| *Patriofelis ulta* | 17505 | AMNH |
| *Pogonodon platycopis* | 6953 | AMNH |
| *Promegantereon ogygia* | B-3-2561 | MNCN |
| *Promegantereon ogygia* | BAT-1-'01-D6-115 | MNCN |
| *Promegantereon ogygia* | BAT-1-'08-D3-4 | MNCN |
| *Promegantereon ogygia* | BAT-1-'08-E3-26 | MNCN |
| *Pseudaelurus* sp. | 62167 | AMNH |
| *Smilodon ensenadensis* | 61 | MCNV |
| *Smilodon fatalis* | LB41 | NMB |
| *Smilodon gracilis* | 69229 | AMNH |
| *Smilodon gracilis* | 69230 | AMNH |
| *Tomarctus* sp. | 67781 | AMNH |
| *Tomarctus* sp. | 67783 | AMNH |
| *Tomarctus* sp. | 67726 | AMNH |
| *Tomarctus* sp. | 67763 | AMNH |
| *Tomarctus* sp. | 67764 | AMNH |
| *Tomarctus* sp. | 67766 | AMNH |
| *Ursus spelaeus* | Jf 1119 | NMB |
| *Ursus spelaeus* | Jf 1120 | NMB |
| **Tibia** |  |  |
| *Aelurodon ferox* | 27479 | AMNH |
| *Aelurodon ferox* | 67459 | AMNH |
| *Amphicyon* sp. | Cast | AMNH |
| *Amphicyon ingens* | 68117-A | AMNH |
| *Amphicyon ingens* | 68117 | AMNH |
| *Amphicyon ingens* | 68122 | AMNH |
| *Amphicyon major* | 10428 | AMNH |
| *Amphicyon major* | 29619 | NHM |
| *Amphicyon* sp. | 18848 | AMNH |
| *Amphicyon* sp. | 26872 | AMNH |
| *Amphicyon* sp. | 617-27298 | AMNH |
| *Amphicyon* sp. | 68104-E | AMNH |
| *Amphicyon* sp. | 68131 | AMNH |
| *Arctodus simus* | 12392 | AMNH |
| *Arctodus simus* | 217-2297 | AMNH |
| *Arctodus simus* | 25531 L | AMNH |
| *Arctodus simus* | 25531 R | AMNH |
| *Barbourofelis fricki* | 61994 | AMNH |
| *Barbourofelis fricki* | 61995 | AMNH |
| *Borophagus* sp. | 23366 | AMNH |
| *Borophagus* sp. | 67637 | AMNH |
| *Borophagus* sp. | 67950-A | AMNH |
| *Borophagus* sp. | 67950 | AMNH |
| *Daphoenodon* sp. | 68276 | AMNH |
| *Daphoenus* sp. | 11857 | AMNH |
| *Epicyon haydeni* | 67414 | AMNH |
| *Epicyon haydeni* | 67418 | AMNH |
| *Epicyon haydeni* | 67616 L | AMNH |
| *Epicyon haydeni* | 67616 R | AMNH |
| *Epicyon saevus* | 67688 | AMNH |
| *Hemicyon* sp. | 68176 | AMNH |
| *Hoplophoneus insolens* | 655 | AMNH |
| *Hyaenodon pervagus* | 19002 | AMNH |
| *Ischyrocyon* sp. | 68153-B | AMNH |
| *Ischyrocyon* sp. | 68157 | AMNH |
| *Machairodus aphanistus* | B-398 | MNCN |
| *Machairodus* sp. | 104726 | AMNH |
| *Machairodus* sp. | M8964 | NHM |
| *Megantereon cultridens* | Se311 | NMB |
| *Paratomarctus euthos* | 61088 | AMNH |
| *Paratomarctus euthos* | 67539 | AMNH |
| *Pogonodon* sp. | 1399 L | AMNH |
| *Pogonodon* sp. | 1399 R | AMNH |
| *Promegantereon ogygia* | BAT-1-'05-F6-42 | MNCN |
| *Promegantereon ogygia* | BAT-1-'06-F4-232 | MNCN |
| *Pseudaelurus* sp. | 62163 | AMNH |
| *Pseudaelurus* sp. | 62173 | AMNH |
| *Smilodon ensenadensis* | 61 | MCNV |
| *Smilodon fatalis* | LB41 | NMB |
| *Smilodon populator* | 2 | SNM |
| *Smilodon* sp. | MRCB-260a | MCNV |
| *Tomarctus* sp. | 67767 | AMNH |
| *Tomarctus* sp. | 67771 | AMNH |
| *Tomarctus* sp. | 67773 | AMNH |
| *Tomarctus* sp. | 67731 | AMNH |
| *Tomarctus* sp. | 67871 | AMNH |
| *Ursus etruscus* | 7531V | MSN |
| *Ursus etruscus* | VA870 | NMB |
| *Ursus spelaeus* | 43822 | NHM |
| *Ursus spelaeus* | Jf1021 | NMB |
| *Ursus spelaeus* | Jf372 | NMB |

Table S4. Detailed description of the anatomical position of each landmark used in this study.

|  | **Pelvis**: |
| --- | --- |
| 1 | Most anterior point of the ventral border of the illium. |
| 2 | Most posterior point of the antero-ventral spine of the ilium. |
| 3 | Point of maximum curvature in the anterior border of the pubis. |
| 4 | Most anterior point of the pubic symphysis. |
| 5 | Most anterior point of the obturator foramen edge. |
| 6 | Most dorsal point of the obturator foramen edge. |
| 7 | Most ventral point of the obturator foramen edge. |
| 8 | Most posterior point of the obturator foramen edge. |
| 9 | Most posterior point of the ischial tuberosity. |
| 10 | Most posterior point of the acetabular incisure. |
| 11 | Point of maximum curvature in the anterior extreme of the acetabular articular surface. |
| 12 | Point of maximum curvature of the internal border of the acetabular articular surface. |
| 13 | Point of maximum curvature in the posterior extreme of the acetabular articular surface. |
| 14 | Most anterior point of the antero-dorsal spine of the ilium. |
| 15 | Most posterior point of the postero-dorsal spine of the ilium. |
| 16 | Most dorsal point of the ischial spine. |
| 17 | Most posterior point of the pubic symphysis. |
|  |  |
|  | **Femur**: |
| 1 | Midpoint of the fovea capitis. |
| 2 | Most posterior point of the lesser trochanter. |
| 3 | Most proximal point of the greater trochanter. |
| 4 | Point of maximum curvature in the proximal edge between the femoral head and the greater trochanter. |
| 5 | Most medial point at the middle of the shaft. |
| 6 | Most posterior point at the middle of the shaft. |
| 7 | Most lateral point at the middle of the shaft. |
| 8 | Proximo-medial corner of the medial condyle. |
| 9 | Proximo-lateral corner of the medial condyle. |
| 10 | Proximo-medial corner of the lateral condyle. |
| 11 | Proximo-lateral corner of the lateral condyle. |
| 12 | Most distal point of the intercondylar fossa. |
| 13 | Most anterior point at the middle of the shaft. |
| 14 | Point of maximum curvature of the lateral edge between the condyle and the trochlea. |
| 15 | Point of maximum curvature of the medial edge between the condyle and the trochlea. |
| 16 | Most proximal point of the lateral edge of the trochlea. |
| 17 | Most proximal point of the medial edge of the trochlea. |
|  |  |
|  | **Tibia**: |
| 1 | Point of maximum curvature of the posterior edge of the medial condyle. |
| 2 | Point of maximum curvature of the posterior intercondylar edge. |
| 3 | Point of maximum curvature of the posterior edge of the lateral condyle. |
| 4 | Most proximal point of the medial condyle. |
| 5 | Point of maximum curvature in the intercondylar eminence. |
| 6 | Most proximal point of the lateral condyle. |
| 7 | Most medial point of the proximal epiphysis. |
| 8 | Most lateral point of the proximal epiphysis. |
| 9 | Most medial point at the middle of the shaft. |
| 10 | Most posterior point at the middle of the shaft. |
| 11 | Most lateral point at the middle of the shaft. |
| 12 | Postero-distal corner of the medial malleolus. |
| 13 | Most distal point of the posterior edge of the distal articular surface. |
| 14 | Most posterior point of the internal edge of the distal articular surface. |
| 15 | Most proximal point of the lateral edge of the distal articular surface. |
| 16 | Midpoint of the medial half of the distal articular surface. |
| 17 | Point of maximum curvature of the lateral edge of the tibial tuberosity. |
| 18 | Point of maximum curvature of the medial edge of the tibial tuberosity. |
| 19 | Most anterior point at the middle of the shaft. |
| 20 | Most distal point of the anterior edge of the distal articular surface. |
| 21 | Point of maximum curvature in the notch of the anterior edge of the distal articular surface. |
| 22 | Antero-distal corner of the medial malleolus. |

Table S5. Stratigraphic ranges and time of divergence for the extinct taxa included in the composite tree used in this paper. The source references for phylogenetic position and stratigraphic range are indicated. The time of divergence of two extinct species (*Arctodus simus* and *Ursus spelaeus*) have been obtained from molecular data (MD). Where the molecular data and the stratigraphic range of the extinct taxa differed, the one with the oldest date have been chosen. Besides, where several nodes overlap at the same date, an arbitrary difference of 0.1 My was introduced between consecutive internal nodes.

| **Taxa** | **Stratigraphic range** | **Ref. for phylogenetic position** | **Ref. for stratigraphic range** |
| --- | --- | --- | --- |
| *Aelurodon ferox* | 15 - 12 my | 2 | 2 |
| *Aelurodon taxoides* | 12 - 9 my | 2 | 2 |
| *Amphicyon* | 23 - 7.2 my | 3 | 4 |
| *Arctodus simus* | MD 5.66 my | 5 | 5 |
| *Barbourofelis* | 11 - 6 my | 6 | 3, 7 |
| *Borophagus* | 12 - 2 my | 2 | 2 |
| *Carpocyon* | 16 - 5 my | 2 | 2 |
| *Daphoenodon* | 23 - 17.5 my | 3 | 3, 7 |
| *Daphoenus* | 39.5 - 27 my | 3 | 3, 7 |
| *Dinictis* | 37 - 26 my | 8 | 7, 8, 9 |
| *Epicyon haydeni* | 10 - 5 my | 2 | 2 |
| *Epicyon saevus* | 7 - 12 my | 2 | 2 |
| *Hemicyon* | 16 - 13.6 my | 10 | 4 |
| *Hoplophoneus* | 37 - 28 my | 8 | 7, 8, 9 |
| *Hyaenodon pervagus* | 34 - 24.6 my | 11 | 12, 13 |
| *Ischyrocyon* | 14 - 8 my | 3 | 3, 7 |
| *Machairodus* | 15 - 2 my | 14 | 15 |
| *Megantereon* | 5.3 - 0.78 my | 14 | 4 |
| *Nimravus* | 34 - 24 my | 8 | 7, 8, 9 |
| *Paratomarctus euthos* | 13 - 9 my | 2 | 2 |
| *Paratomarctus temerarius* | 16 - 13 my | 2 | 2 |
| *Patriofelis* | 50.3 - 40.4 my | 11 | 4 |
| *Pogonodon* | 34 - 23 my | 8 | 7, 8, 9 |
| *Promegantereon ogygia* | 11 - 8.2 my | 16 | 13 |
| *Pseudaelurus* | 20.4 - 4.9 my | 17 | 4 |
| *Smilodon* | 4.9 - 0.01 my | 14 | 4 |
| *Tomarctus* | 16 - 14 my | 2 | 2 |
| *Ursus etruscus* | 2.6 - 1.3 my | 10 | 13 |
| *Ursus spelaeus* | MD 2.75 my | 5 | 5 |
|  |  |  |  |
|  | **Time of divergence** |  | **Ref. for time of divergence** |
| Order Creodonta | 65.1 my | 11 | 18 |
| Family Amphicyonidae | 61.5 my | 19 | 18 |
| Family Barbourofelidae | 20 my | 6 | 4 |
| Family Nimravidae | 37 my | 8 | 7, 8, 9 |
| Subfamily Borophaginae | 34 my | 2 | 2 |
| Subfamily Machairondontinae | 15 my | 14 | 15 |

REFERENCES

1. Samuels JX, Meachen JA, Sakay SA: **Postcranial morphology and the locomotor habits of living and extinct carnivorans**. *J Morphol* 2013, **274**:121-146.

2. Wang XM, Tedford RH, Taylor BE: **Phylogenetic sytematics of the Borophaginae (Carnivora: Canidae)**. *Bull Am Mus Nat Hist* 1999, **243**:1-391.

3. Hunt RM: **Amphicyonidae**. In: *Evolution of Tertiary mammals of North America: Volume 1, terrestrial carnivores, ungulates, and ungulate like mammals*. Edited by Janis CM, Scott KM, Jacobs LL. Cambridge: Cambridge University Press; 1998:196-227

4. Paleobiology database. http://fossilworks.org/bridge.pl

5. Krause J, Unger T, Noçon A, Malaspinas AS, Kolokotronis SO, Stiller M, Soibelzon L, Spriggs H, Dear PH, Briggs AW, Bray SCE, O'Brien SJ, Rabeder G, Matheus P, Cooper A, Slatkin M, Pääbo S, Hofreiter M: **Mitochondrial genomes reveal an explosive radiation of extinct and extant bears near the Miocene-Pliocene boundary**. *BMC Biol* 2008, **8**:220.

6. Morlo M, Peigne S, Nagel D: **A new species of *Sansanosmilus*: implications for the systematic relationships of the family Barbourofelidae new rank (Carnivora, Mammalia)**. *Zool J Linn Soc* 2004, **140**:43-61.

7. Janis CM, Gunnell G, Uhen M: *Evolution of Tertiary Mammals of North America Vol. 2: Small Mammals, Edentates, and Marine Mammals*. Cambridge, UK: Cambridge University Press; 2008.

8. Peigné S: **Systematic review of European Nimravinae (Mammalia, Carnivora, Nimravidae) and the phylogenetic relationships of Palaeogene Nimravidae**. *Zool Scr* 2003, **32**:199-229.

9. Martin LD: **Nimravidae**. In: *Evolution of Tertiary mammals of North America: Volume 1, terrestrial carnivores, ungulates, and ungulate like mammals*. Edited by Janis CM, Scott KM, Jacobs LL. Cambridge: Cambridge University Press; 1998:228-235.

10. McLellan B, Reiner DC: **A review of Bear evolution**. In: *Bears: Their Biology and Management, Vol. 9, Part 1: A Selection of Papers from the Ninth International Conference on Bear Research and Management*. Missoula, Montana; 1994:85-96.

11. Gunnell GF: **Creodonta**. In: *Evolution of Tertiary mammals of North America: Volume 1, terrestrial carnivores, ungulates, and ungulate like mammals*. Edited by Janis CM, Scott KM, Jacobs LL. Cambridge: Cambridge University Press; 1998:91-109.

12. Nagel D, Morlo M: **Guild structure of the carnivorous mammals (Creodonta, Carnivora) from the Taatsiin Gol area, Lower Oligocene of Central Mongolia**. *Deinsea* 2003, **10**:419-429.

13. NOW Database. http://www.helsinki.fi/science/now/index.html

14. Anton M, Salesa MJ, Morales J, Turner A: **First known complete skulls of the scimitar-toothed cat *Machairodus aphanistus* (Felidae, Carnivora) from the Spanish late Miocene site of Batallones-1**. *J Vertebr Paleontol* 2004, **24**:957-968.

15. Turner A, Anton M: *The big cats and their fossil relatives*. New York, US: Columbia University Press; 1997.

16. Salesa MJ, Antón M, Turner A, Morales J: **Functional anatomy of the forelimb in *Promegantereon ogygia* (Felidae, Machairodontinae, Smilodontini) from the Late Miocene of Spain and the origins of the sabre-toothed felid model**. *J Anat* 2010, **216**:381-396.

17. Rothwell T: **Phylogenetic systematics of Northamerican *Pseudaelurus* (Carnivora, Felidae)**. *Am Mus Nov* 2003, **3403**:64.

18. Nyakatura K, Bininda-Emonds ORP: **Updating the evolutionary history of Carnivora (Mammalia): a new species-level supertree complete with divergence time estimates**. *BMC Biol* 2012, **10**:12.

19. Finarelli JA, Flynn JJ: **Ancestral state reconstruction of body size in the Caniformia (Carnivora, Mammalia): the effects of incorporating data from the fossil record**. *Syst Biol* 2006, **55**:301-313.
