# Supplementary material for: A three-dimensional analysis of the morphological evolution and locomotor behaviour of the carnivoran hind limb
Source: BMC Evol Biol. 2014 Jun 14;14:129. doi: 10.1186/1471-2148-14-129 (PMC4065579; doi:10.1186/1471-2148-14-129)
Supplement: Additional file 6 — Nexus file of the composite tree used in this paper. [file 1471-2148-14-129-S6.pdf]

```
#NEXUS

begin TREES;

	TRANSLATE
		1 Daphoenus,
		2 Amphicyon,
		3 Ischyrocyon,
		4 Daphoenodon,
		5 Tomarctus,
		6 Aelurodon_ferox,
		7 Aelurodon_taxoides,
		8 Paratomarctus_euthos,
		9 Paratomarctus_temerarius,
		10 Carpocyon,
		11 Epicyon_haydeni,
		12 Epicyon_saevus,
		13 Borophagus,
		14 Nasua_nasua,
		15 Bassariscus_astutus,
		16 Potos_flavus,
		17 Procyon_lotor,
		18 Meles_meles,
		19 Eira_barbara,
		20 Lontra_canadensis,
		21 Ailurus_fulgens,
		22 Urocyon_cinereoargenteus,
		23 Vulpes_lagopus,
		24 Vulpes_velox,
		25 Vulpes_vulpes,
		26 Nyctereutes_procyonoides,
		27 Otocyon_megalotis,
		28 Cerdocyon_thous,
		29 Chrysocyon_brachyurus,
		30 Speothos_venaticus,
		31 Canis_adustus,
		32 Canis_mesomelas,
		33 Lycaon_pictus,
		34 Cuon_alpinus,
		35 Canis_simensis,
		36 Canis_aureus,
		37 Canis_latrans,
		38 Canis_lupus,
		39 Acinonyx_jubatus,
		40 Puma_concolor,
		41 Leptailurus_serval,
		42 Lynx_rufus,
		43 Neofelis_nebulosa,
		44 Uncia_uncia,
		45 Panthera_tigris,
```

46 Panthera_onca,
47 Panthera_pardus,
48 Panthera_leo,
49 Hyaena_hyaena,
50 Hyaena_brunnea,
51 Crocuta_crocuta,
52 Machairodus,
53 Promegantereon_ogygia,
54 Megantereon,
55 Smilodon,
56 Barbourofelis,
57 Pseudaelurus,
58 Hoplophoneus,
59 Nimravus,
60 Pogonodon,
61 Dinictis,
62 Ursus_arctos,
63 Ursus_maritimus,
64 Ursus_spelaeus,
65 Ursus_etruscus,
66 Ursus_americanus,
67 Ursus_thibetanus,
68 Melursus_ursinus,
69 Helarctos_malayanus,
70 Tremarctos_ornatus,
71 Arctodus_simus,
72 Ailuropoda_melanoleuca,
73 Hemicyon,
74 Patriofelis,
75 Hyaenodon_pervagus;

TREE 'Untitled Tree++' =
((74:9.9,75:25.7):14.8,((((49:1.5,50:1.5):1.5,51:3.0):50.0,((59:13.4,(58:9.3,(60:14.2,61:11.2):0.1):0.1):0.1,(56:14.5,(57:15.5,((52:13.1,(53:2.8,(54:4.5,55:5.29):4.7):5.1):1.3,((((39:7.0,40:7.0):4.5,42:11.5):4.1,41:15.6):0.7,(43:9.2,((44:3.5,45:3.5):2.6,(46:3.9,(47:2.9,48:2.9):1.0):2.2):3.1):7.1):0.1):4.0):0.1):17.0):15.5):12.0,(((1:12.5,4:22.0):0.1,(2:15.8,3:15.0):16.6):21.9,(((21:35.0,(((20:14.7,19:14.7):2.3,18:17.0):14.7,(16:22.0,(14:12.1,(15:12.0,17:12.0):0.1):9.9):9.7):3.3):13.0,(73:3.6,(72:17.1,((70:5.66,71:5.56):9.84,(68:6.4,((69:4.8,(66:3.8,67:3.8):1.0):0.1,(65:1.45,(64:2.62,(62:1.0,63:1.0):1.75):0.1):2.05):1.5):9.1):1.6):0.1):30.8):13.4,(((5:2.0,(6:3.0,7:6.0):1.0):0.2,((8:7.0,9:3.0):0.1,(10:11.0,(13:10.0,(11:7.0,12:5.0):0.1):3.9):0.1):0.1):17.8,(22:16.3,((27:16.0,(26:15.9,(25:5.8,(23:3.0,24:3.0):2.8):10.1):0.1):0.1,((33:6.6,30:6.6):2.7,((28:9.0,29:9.0):0.1,((31:4.2,32:4.2):4.8,(34:4.1,(35:3.9,((36:2.4,37:2.4):0.2,38:2.6):1.3):0.2):4.9):0.1):0.2):6.8):0.2):17.7):27.4):0.1):3.5):0.1):21.2;
end;
